# Looking back to look ahead: the temporal dimension of conservation seed bank collections

**DOI:** 10.1111/nph.70187

**Published:** 2025-05-06

**Authors:** Efisio Mattana, Sandrine Godefroid, Stephanie Miles, Angelino Carta, Andreas Ensslin, Ted Chapman, Juan Viruel

**Affiliations:** ^1^ Royal Botanic Gardens, Kew Wakehurst Ardingly RH17 6TN UK; ^2^ Meise Botanic Garden Meise 1860 Belgium; ^3^ Department of Biology University of Pisa Pisa 56126 Italy; ^4^ CIRSEC – Centre for Climate Change Impact University of Pisa Pisa 56126 Italy; ^5^ Geneva Botanical Garden Geneva 1260 Switzerland; ^6^ Royal Botanic Gardens, Kew Kew Gardens Richmond TW9 3DS UK

**Keywords:** climate change, ecological restoration, *ex situ* conservation, genetic diversity, plant adaptation, plant evolution, resurrection approach, seed conservation

## Abstract

A wealth of plant material and data is stored globally in conservation seed banks. This material represents not only a repository of plant genetic resources but also an asset for nature‐based solutions (NbS), such as ecological restoration and reforestation, and research in plant science. Here, we explore the temporal and spatial dimensions of seed collections and the challenges limiting their use in NbS and research, while highlighting how they could be a source of material for adaptation and evolution studies. However, existing seed lots originally collected for conservation purposes will not be sufficient to support NbS and research on their own. We propose a long‐term experimental approach that, together with new targeted collecting programmes, can leverage the temporal dimension of seed collections by carrying out repeated sampling from the same population. At the same time, we stress how these approaches will benefit from new dedicated collections holding seeds from each maternal line separately. By moving towards a bidimensional (space and time) collecting approach, conservation seed banks can go beyond long‐term conservation per se and transform their collections into dynamic repositories capable of addressing pressing ecological, evolutionary, and conservation questions and help to understand and shape plant communities of the future.

## Introduction

Seed banks have historically served as tools for the preservation of agricultural crops (Linington, 2003). Over time, their scope has expanded to encompass the *ex situ* conservation of wild plant species, aligning with international biodiversity initiatives such as the Convention on Biological Diversity and its Global Strategy for Plant Conservation (https://www.cbd.int/convention/text). Seed conservation technologies and practices were originally limited to the Global North. However, they have since been widely adopted worldwide (Hay & Probert, 2013), bolstered by standards from Bioversity International (formerly IPGRI: International Plant Genetic Resources Institute) and FAO (FAO/IPGRI, 1994) and prominent projects, such as the network of gene banks managed by the Alliance Bioversity & CIAT (https://alliancebioversityciat.org) and the Millennium Seed Bank Project (now Partnership; MSBP), managed by the Royal Botanic Gardens, Kew (https://www.kew.org/science/our‐science/projects/banking‐the‐worlds‐seeds). While seed banks generally prioritize threatened species, they also focus on the conservation of species with a restricted geographical distribution (e.g. endemics) and/or wild species of potential economic importance, such as crop wild relatives, or species traditionally used by humans (e.g. the 3Es strategy adopted by the MSBP: endangered, endemic, and economic; Griffiths *et al*., 2015, Liu *et al*., 2018). Thus, seed banks now represent a cornerstone of biodiversity conservation, contributing to the achievement of Target 4 of The Kunming‐Montreal Global Biodiversity Framework (https://www.cbd.int/doc/decisions/cop‐15/cop‐15‐dec‐04‐en.pdf).

Seed banks rely on, and amplify, the natural properties of seeds of most species to travel and remain viable over space and time, when the right storage conditions of temperature and humidity are provided. Their success in plant conservation arises from three major factors (Li & Pritchard, 2009): (1) the economic feasibility of long‐term storage; (2) the efficiency of preserving genetic diversity in minimal physical space; and (3) the applicability of standardized protocols across diverse taxonomic groups. Desiccation‐tolerant seeds, which represent the majority of species worldwide, can be dried to remove excess water and stored below freezing temperatures for long‐term conservation (Roberts, 1973). Globally, *c*. 8% of species are estimated to produce desiccation‐sensitive seeds, though this figure increases to 18.5% in tropical and subtropical moist broadleaf forests (Wyse & Dickie, 2017). For these seed desiccation‐sensitive species and those for which conventional seed banking is also not applicable (the so‐called ‘exceptional species’), alternative conservation measures should be applied, depending on the specific constraints that limit or preclude their storage in conventional seed banks (Walters & Pence, 2021; Pence *et al*., 2022). More than five decades of intensive global seed collection programmes have created an unparalleled repository of plant material and associated metadata. For example, the MSBP holds over 2.4 billion seeds from nearly 40 000 species (https://www.kew.org/science/collections‐and‐resources/collections/seed‐collection), while a recent European survey revealed that more than 150 000 accessions representing over 12 000 taxa are conserved across 100 seed banks in 29 countries (A. Ensslin *et al.*, pers. comm.; see also Carta *et al*., 2025).

The significance of seed banks extends beyond conserving species genetic diversity. They also provide critical resources to counteract biodiversity loss and anthropogenic climate change, two complex and interlinked phenomena that perpetuate each other through a feedback loop (Pörtner *et al*., 2021). Conservation seed banks can contribute to breaking this cycle by preserving genetic resources that are the main asset for species reintroduction (White *et al*., 2023), supporting ecological restoration and reforestation, by providing limited quantities of seeds or seedlings of native species (for instance for seed production by nurseries) and information on their seed biology and germination ecology (Merritt & Dixon, 2011; Kildisheva *et al*., 2020; Goodale *et al*., 2023) and research under a changing climate (Havens *et al*., 2015; Carta *et al*., 2022; Mattana *et al*., 2022). However, to move beyond long‐term *ex situ* conservation of genetic resources of threatened species per se, and properly support NbS while tracking within‐species genetic and functional variation and evolution under a fast‐changing climate, a transformative change towards a bidimensional (i.e. space and time) approach is required, connecting seed bank practices with the natural processes regulating such variation (Merritt & Dixon, 2011; Etterson *et al*., 2016; Martyn Yenson *et al*., 2021; Rauschkolb *et al*., 2022a).

In this Viewpoint, we outline the importance of the spatial and temporal dimensions of seed bank collections in supporting NbS and research in plant science. We explore how seed collections can better capture the evolutionary and adaptive responses of plant populations to environmental changes and highlight the risks of temporal genetic maladaptation when using stored seed collections in restoration efforts under current and future climates, and how conservation seed banks can optimize seed collecting protocols to ensure both spatial and temporal representation of genetic diversity. We argue that leveraging the temporal dimension, through repeated, systematic collections from the same populations, offers unique opportunities to investigate evolutionary dynamics and climate change adaptability in plant species, taking into account the potential for evolution in species responses to climate change (Nadeau & Urban, 2019). We propose an experimental approach and protocols for seed banking practices that integrate these dimensions. By integrating these approaches, conservation seed banks can transform existing collections into dynamic resources for addressing pressing ecological, evolutionary, and conservation questions.

## Looking back: seed lots stored in seed banks for conservation purposes

**Fig. 1:**
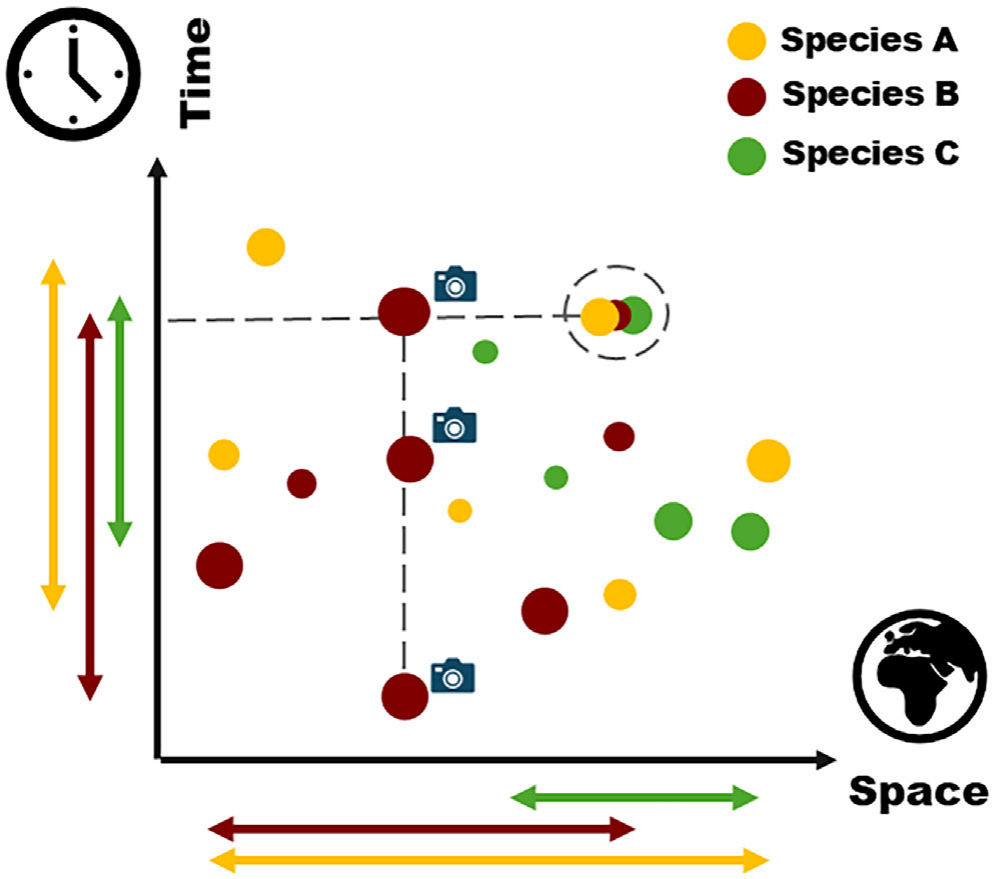
Bidimensional representation of seed collections stored in seed banks. The position of the collections of a species along the Space axis provides information on the geographical (and potentially genetic) representativeness of the stored collection with respect to the natural distribution of the species. Distribution along the Time axis indicates when a collection was made, providing a genetic snapshot of a population at a specific point in time, and the time span that is covered by the collections of a species. By combining the two distributions, it is possible to identify populations for which multiple collections were carried out over time (vertical dashed line), providing a potential source for studying plant adaptation and evolution *sensu* Franks *et al*. (2018), as well as populations of different species collected at the same time (dashed horizontal line) and in the same area (dashed circle), which could constitute baseline points for future comparative studies (Franks *et al*., 2018; Everingham *et al*., 2021; Rauschkolb *et al*., 2022b). The size of the points represents a proxy for the size of the collections. The horizontal wide arrows represent the collections' geographical range, while the vertical ones the collections' time span for each species.

Seed collections stored in conservation seed banks vary significantly in their origin (i.e. geographical location), size (i.e. number of sampled individuals and stored viable seeds), and age (i.e. time since collecting), which can be summarized within a two‐dimensional conceptual framework along a spatial and a temporal dimension (Fig. 1). For each target species, the spatial dimension represents the geographical range, the population(s) distribution, and – by extension – the potential intraspecific genetic diversity captured by the collections (Gargiulo *et al*., 2025).

The temporal dimension indicates the time span covered by the collections of a species and, when multiple collections are available from the same population, provides insight into the temporal genetic dynamics of a population, that is, into its evolution (Fig. 1). Together, these dimensions enable not only the identification of genetic representation and evolutionary potential in species but also the study of evolution in action, providing a unique framework for leveraging genetic resources for NbS (Franks *et al*., 2018; Everingham *et al*., 2021; Rauschkolb *et al*., 2022b).

### The space dimension

Determining what, how much, and from where to collect has long been central to effective *ex situ* conservation (Smith *et al*., 2016; Rivière *et al*., 2018; Hoban, 2019). Guidelines for the effective conservation of genetic diversity have been produced and have evolved through the years (see Whitlock *et al*., 2016 for a summary). Collecting sites should cover the geographical and ecological distribution of the species, aiming to capture all potential genetic variation, with the number of sites to be sampled varying according to the purpose of the collection, seed availability, mating system, and other factors that influence genetic diversity (Loveless & Hamrick, 1984; Duminil *et al*., 2009; Martyn Yenson *et al*., 2021). Population genetic structure should be considered, and seed collection plans should incorporate spatial considerations, particularly for poorly connected taxa (Hoban & Schlarbaum, 2014; White *et al*., 2025). This is particularly important since genetic diversity is now being specifically targeted in the goals and targets of the Kunming‐Montreal Global Biodiversity Framework (Hoban *et al*., 2023).

Seed collecting sampling strategies affect not only *ex situ* conservation per se but also the potential use of stored collections in NbS. Emerging strategies have moved beyond the ‘local is best’ paradigm, which assumed that seeds from local populations perform better in restoration projects. This assumption holds in many cases, although the frequency and scale of this response may be site, population, and/or species‐specific (Leimu & Fischer, 2008; Hereford, 2009; Bucharova *et al*., 2017). However, recent evidence highlights that reduced genetic diversity in small local populations and environmental disturbances, including climate change and habitat fragmentation, are major challenges for the long‐term persistence of plant populations and proposes identifying genetically diverse climate‐smart source populations, more adapted to future climates for conservation and use in NbS (Breed *et al*., 2013; Jones, 2013; Broadhurst *et al*., 2015; Havens *et al*., 2015; Martyn Yenson *et al*., 2021; St.Clair *et al*., 2022; Jordan *et al*., 2024). Identifying such populations requires integrating genetic and ecological data to match restoration needs, with source materials likely to perform well under changing climates (Laccetti *et al*., 2024). Integrating spatial considerations into seed collection planning, particularly for species with limited gene flow, ensures that conservation seed banks maintain a representative and genetically diverse repository (Hoban & Strand, 2015). These efforts also support research and restoration activities, providing the genetic resources needed to address biodiversity loss and enhance ecosystem resilience in the face of climate change.

### The time dimension

Seed lots stored in conservation seed banks capture a genetic snapshot of a population at a specific point in time (Fig. 1), preserving the environmental and genetic contexts of their collection period. As natural populations continue to evolve in response to changing environmental and climatic conditions, the genetic composition of these populations may diverge significantly from the preserved seed lots (Hamilton, 1994). The phenotypic traits shaped by past environmental conditions are retained in the banked seeds, representing the ‘genetic memory’ of their collection time. Meanwhile, shifts in environmental optima drive adaptation in the wild, potentially resulting in the banked collections becoming genetically distinct from their present‐day counterparts (Hamilton, 1994; Schoen & Brown, 2001). Traits associated with phenology (e.g. germination, budding, flowering, seed ripening) have been found to evolve particularly rapidly under changed natural or artificial selection processes (Franks *et al*., 2007; Ensslin *et al*., 2018; Rauschkolb *et al*., 2023). Such divergence could be more pronounced with life history traits that influence gene flow between populations or generation times, such as the mating system or the distinction between annuals and perennials. Species with shorter generation times, such as annuals, can adapt more rapidly to environmental changes through multiple generational turnovers within a shorter period (Duminil *et al*., 2009). By contrast, perennials with longer generation times evolve more slowly but may exhibit greater stability in response to environmental perturbations (Loveless & Hamrick, 1984). Plants germinated from old banked collections may thus harbour traits or genotypes that are maladaptive, *sensu* Brady *et al*. (2019), under current and/or future environmental conditions, raising challenges when considering their use in restoration or other NbS. This temporal differentiation should be carefully evaluated by seed banks to ensure that conserved collections remain as relevant and beneficial as possible for conservation and restoration efforts (Vitt *et al*., 2004; Havens *et al*., 2015).

Despite the importance of these temporal considerations, there has been limited focus on building sequential collections that track evolutionary changes over time within the same populations (see Case Study 1). However, when available, such temporal series offer unique opportunities to explore plant evolution, adaptation, and responses to climate change. The shift in genetic diversity that can develop over time between banked seed lots and seeds collected in the wild makes the former perfect candidates for experimental evolution studies applying a resurrection ecology approach (Franks *et al*., 2018; Nadeau & Urban, 2019). Available old seed lots can be used in comparative studies with recent new collections made from the same natural populations (see Case Study 2). Investigating the genetic differentiation between samples collected in the same locality across time allows for the understanding of which plant species and which life history traits are most subject to change. In addition, conservation seed banks usually store seed lots from a phylogenetically wide range of species, making possible large‐scale studies on parallel evolution (Rauschkolb *et al*., 2023). Both case studies underscore the untapped potential of leveraging temporal dimensions in seed banking for both ecological and evolutionary studies, providing critical insights into plant responses to climate change and other environmental drivers.

### Case Study 1: temporal dimension of the UK flora collections stored at the MSB

**Fig. 2:**
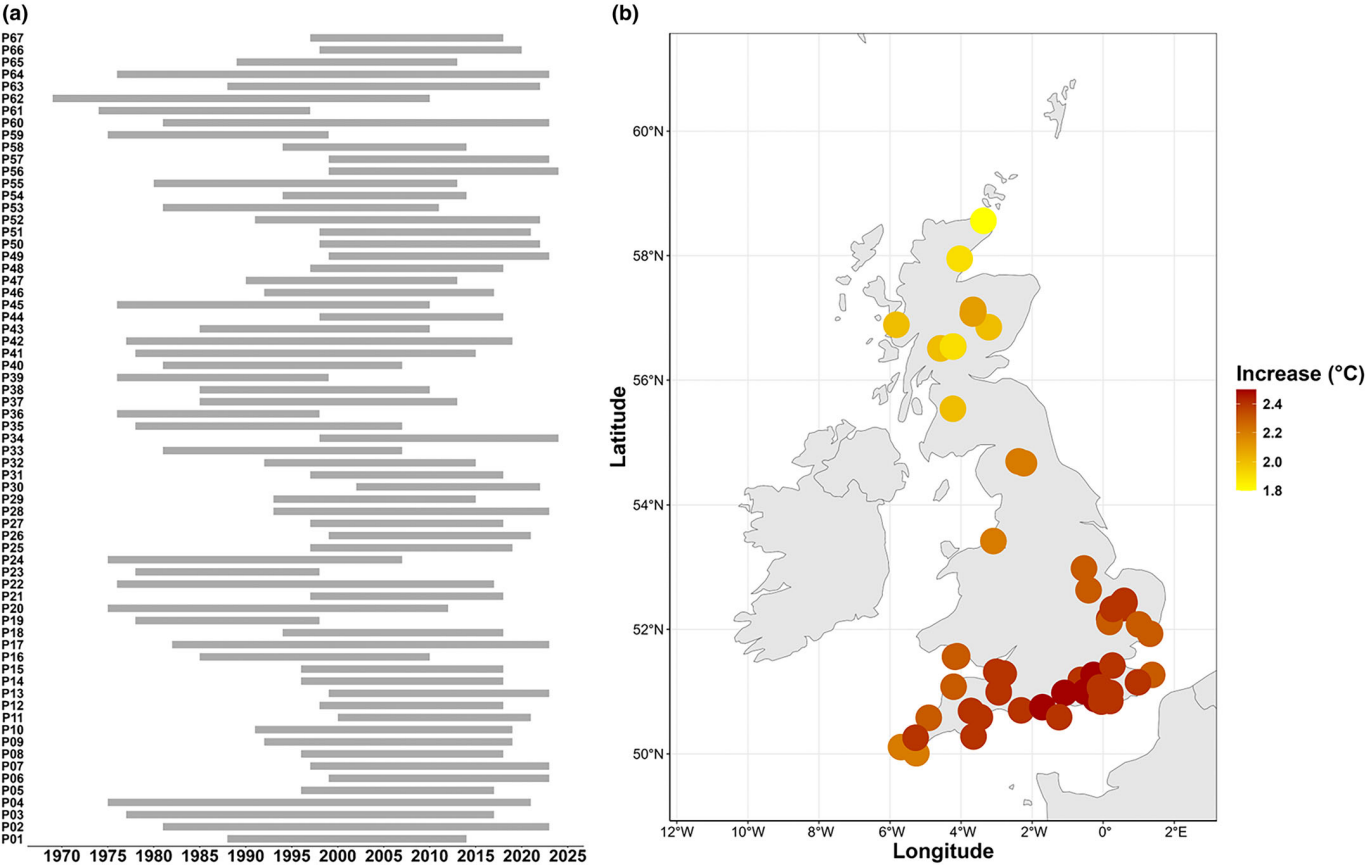
Temporal repeated collections of the UK flora stored at the Millennium Seed Bank. (a) Timespan of repeated collections from the same UK population (within 1 km distance) with a lag of at least 20 yr. (b) Distribution of the populations with repeated sampling. Colour of each point represents the increase in mean annual temperature predicted for the period 2071–2100, according to the most optimistic IPCC SSP1‐2.6 Sustainable development scenario. Climatic data were downloaded from the chelsa website v.2.1 (Karger *et al*., 2017, 2021). Future climatic data correspond to the CMIP6 ISIMIP3 UKESM1‐0‐LL model of the UK Met Office Hadley Centre (Tang *et al*., 2019).

The UK's flora comprises *c*. 9000 vascular plant taxa, 34% of which are considered native and is among the best‐documented globally (BSBI, 2020). The Millennium Seed Bank (MSB) has been collecting seeds from the UK flora since the late 1960s and it now holds 77% of the native and archaeophyte flora, and 87% of all threatened taxa listed in the GB Red List for Vascular Plants (Cheffings *et al*., 2005). However, the MSB's early focus on maximizing species coverage, and more recently on enhancing intraspecific diversity across ecological gradients, has resulted in limited temporal replicates of the same populations. Repeated collections with a significant time lag of at least 20 yr are available for only 67 populations from 60 taxa (Supporting Information Table S1). These collections are concentrated in the southern half of the United Kingdom, and most are limited to two sampling events (Fig. S1; Table S1), with nine species with at least one population having sampling covering 40 yr or more (Fig. 2a; Table S1).

These limited temporal series demonstrate the inevitable trade‐offs between prioritizing spatial vs temporal coverage within the constrained resources available for seed collecting and banking. Yet, they also highlight the potential value of historical collections for investigating evolutionary responses to environmental change. Populations in the South of England are predicted to have a higher increase in mean annual temperature among those identified, highlighting the potential value of these collections in studies of plant evolution and adaptation vs climate change (Fig. 2b).

### Case Study 2: resurrection studies using seeds stored at Meise Botanic Garden

Meise Botanic Garden (Meise BG) began storing seeds of the Belgian flora in the late 1980s. The aim was to conserve threatened species as a priority. As a result, the majority of collections currently available in the seed bank consist of red‐listed species. When the institution was asked to provide seeds for resurrection ecology studies, it quickly became apparent that the list of taxa that could be used for such experiments was going to be limited, given all the required criteria: (1) target species with a short life cycle (as they are more likely to show rapid evolution); (2) target species with sufficiently large surviving wild populations to enable sustainable recollection; (3) accessions stored for at least 20 yr to give time for evolutionary processes to be detected; (4) accessions should have a precise sampling date and location records (with older collections often being made before GPS technology was available); and (5) populations located in nature reserves in order to decrease the probability of local extinction. However, in addition to threatened species, Meise BG had previously collected several nonred‐listed taxa in order to allow quality collections from large populations. This strategy proved to be valuable for the resurrection ecology studies, as it allowed several nonthreatened taxa to qualify for the experiments. Ultimately, only 16 accessions from 14 taxa could be selected and compared with recent collections from the same location, representing only 1.6% of the collections stored at Meise BG.

The resurrection experiments involved growing ancestor plants (derived from stored seeds) and comparing them with their contemporary descendants collected from the same location. Comparing ancestors raised from these stored seeds with their contemporary descendants under common conditions has given rise to some pioneering outcomes. For instance, descendant plants were generally taller than their ancestors under well‐watered conditions, but smaller under drought, suggesting some adaptation to increased drought (Rauschkolb *et al*., 2022a). Descendants also exhibited faster growth and earlier flowering than their ancestors (Rauschkolb *et al*., 2022b). These phenological shifts correlated with increasing aridity at the original collection sites. Earlier flowering is hypothesized to confer an adaptive advantage under drought conditions by reducing water demand during the critical reproductive phase (Karitter *et al*., 2024). Descendants produced more vegetative biomass when grown in competition with other plants, suggesting evolutionary selection for increased competitiveness in resource‐scarce environments (Karitter *et al*., 2025). When descendants and ancestors were transplanted back to their original sites, descendants showed lower mortality, faster germination, and larger overall plant size, further supporting their enhanced adaptation to contemporary environmental conditions (Karitter *et al*., 2024).

These examples show that using seed collections, already available in conservation seed banks in a resurrection approach, has immense potential for studying evolutionary responses to environmental change. They demonstrate how stored seeds can serve as a time capsule, enabling direct comparisons across generations. However, these studies suffer from some drawbacks that reduce the robustness of the results, such as the fact that relatively few taxa could be studied, with only one population per species, and stored seed lots were bulked (i.e. not collected by maternal line). While it is possible to use existing seed collections for resurrection studies, it would be preferable for future collecting strategies in at least some species to be explicitly designed to address these constraints.

### Challenges in using stored seed lots for NbS and research

While conservation seed banks represent an unparalleled resource for genetic material and associated data for NbS (Liu *et al*., 2018; White *et al*., 2023), and for studies in plant auto‐ and macroecology and evolution (Carta *et al*., 2022; Mattana *et al*., 2023; Rauschkolb *et al*., 2023), several challenges can limit the use of their collections for ecological restoration and scientific research at the species level. These challenges primarily arise from limitations in seed lot size, spatial and temporal coverage, seed longevity, and the management of species genetic diversity within collections.

#### Seed lot size

Seed lot size significantly impacts the utility of collections for restoration and research purposes. Conservation seed banks usually deal with collections comprising relatively small seed lots compared to agricultural or forestry gene banks, which often deal with large‐scale materials. In the Geneva seed bank, for instance, only one‐third of seed lots exceed 5000 seeds, which is the minimum size recommended by the European Native Seed COnservation NETwork guidelines (ENSCONET, 2009). The relatively small size of seed lots in conservation seed banks prompted the development of the concept of restoration seed banks: ‘conservation centres able to provide large seed supplies with high genetic diversity and with the expertise to improve seed processing and germination’ (Merritt & Dixon, 2011). However, it is not a matter of seed supply only. The size of a collection can also affect the number and kind of experiments that can be carried out on them (and therefore, the data that can be gathered) or the potential to share material for research or other purposes (Way, 2003; Martyn Yenson *et al*., 2021). In addition, small seed lots are quickly depleted by continuous use, undermining their value as long‐term *ex situ* conservation collections. Seed lot size also affects the amount of genetic diversity stored in each collection as well as the number of maternal lines represented (to be described later).

#### Spatial and ecological representativeness

In Europe, the large majority of species are typically represented with less than five different populations in seed banks (Carta *et al*., 2025), which is a minimum recommendation for seed banks (ENSCONET, 2009). This shortfall could arise from logistical and financial constraints, incomplete knowledge of a species' distribution, or prioritization of unbanked species over repeated collections from well‐represented taxa (Clubbe *et al*., 2020). Seed banks are required to weigh these factors against the need for more comprehensive multi‐population sampling required to represent a species' full geographic and ecological range and ensure collections encompass the full spectrum of genetic diversity, particularly for species with fragmented populations or restricted distributions (Hoban & Schlarbaum, 2014).

#### Temporal limitations and seed longevity

Stored seeds age, and they do it at different paces as species vary in their longevity under seed banking conditions (Ellis, 1991; Hay *et al*., 2022). This is particularly problematic for short‐lived species, which lose viability after a short amount of time under cold storage (Walters & Pence, 2021). This variability in longevity is a challenge for the management of seed bank collections and highlights the need for regular monitoring protocols to maintain the utility of stored seeds for restoration and research purposes (De Vitis *et al*., 2020). Furthermore, even when a short ‘shelf life’ is not a problem, older seed lots may no longer align with current conservation priorities or ecological requirements, as they represent genetic and environmental conditions from decades past. As shown in the MSB's UK flora analysis (see Case Study 1), very few populations are usually sampled repeatedly over time, limiting the availability of historical series necessary for tracking evolutionary and adaptive changes.

#### Maternal lines separation

Maintaining separate maternal lines (i.e. the material collected from different individuals of the same population) is an effective way to increase the value of seed collections for multiple applications (van der Merwe *et al*., 2023), such as fine‐scale genetic studies, studies on evolutionary potential, precise analyses of population structure, and propagation of genetically diverse populations for conservation and restoration purposes. Although international guidelines recommend keeping maternal lines separate (Center for Plant Conservation, 2019; Yenson *et al*., 2021), bulk sampling remains the norm in practice (Etterson *et al*., 2016). While this approach simplifies collection, storage, and curatorial procedures and significantly reduces costs, it also compromises the genetic resolution and utility of the collections. Notable exceptions include UK tree species (Kallow & Trivedi, 2017; Clubbe *et al*., 2020), and highly threatened populations stored at the MSB, for which maternal lines are stored separately.

## Looking ahead: towards fit‐for‐NbS‐and‐research seed collections

Addressing the challenges related to the use of available stored collections requires a forward‐looking approach that integrates advances in conservation biology and restoration science. By adopting innovative practices, such as dynamic seed banking and genetic monitoring, conservation seed banks can enhance their capacity to support NbS and respond to the challenges posed by climate change and biodiversity loss. They must evolve to meet the growing demand for genetic resources in ecological restoration, research, and climate adaptation initiatives. Current collecting practices often prioritize breadth over depth (i.e. the spatial over the temporal dimension of seed collections), focusing on maximizing taxonomic coverage while overlooking the potential of repeated and targeted collections. A forward‐thinking approach should integrate spatial, temporal, and biological dimensions to create seed collections optimized for NbS and evolutionary research.

### Experimental approach

**Fig. 3:**
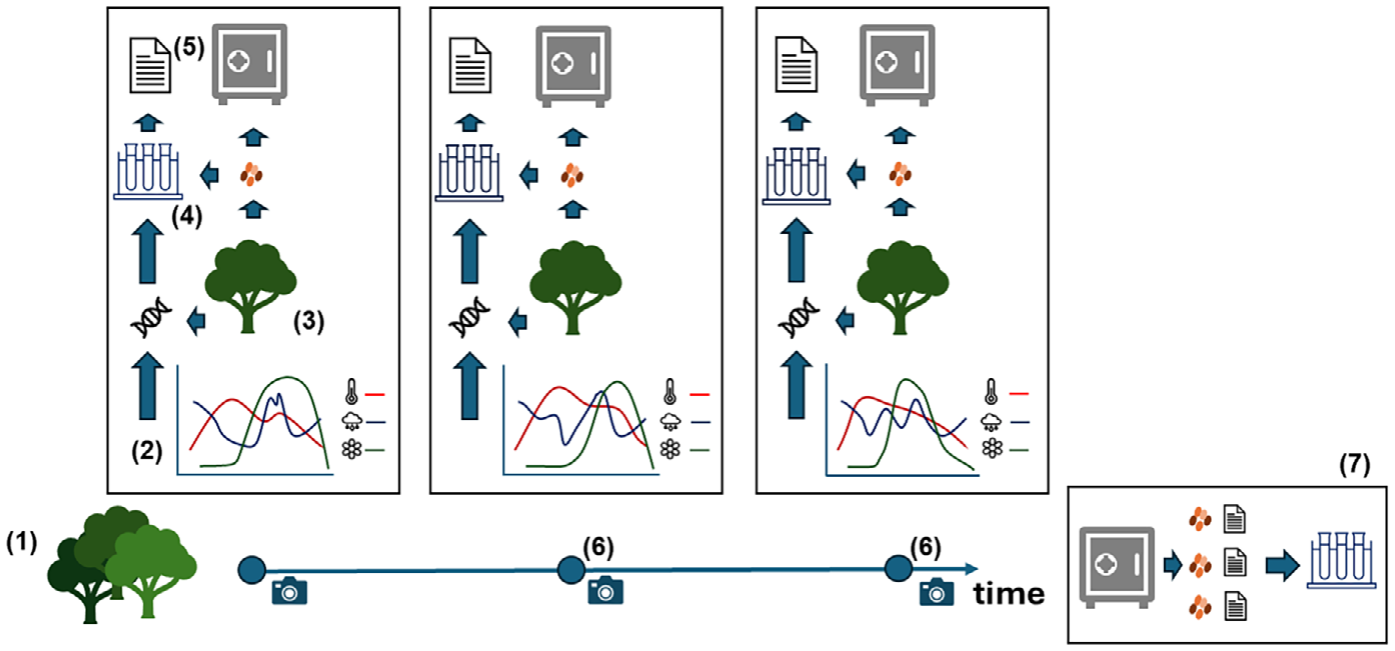
Experimental steps for creating historical series of seed collections available for future studies in plant adaptation and evolution. (1) Long‐term population management: identify populations for which long‐term conservation and monitoring can be reliably maintained. (2) Environmental monitoring: collect detailed environmental data (e.g. rainfall, temperature) and phenological observations (e.g. flowering and fruiting times) for each collection site. (3) Maternal line separation: collect seeds from marked individual plants (for woody species) or plots (for annual or permanent herbs), ensuring that maternal lines remain distinct to preserve trackable genetic diversity. Leaf tissue from each mother plant is collected and preserved in silica gel to allow estimation of the genetic diversity of the original population at the time of the collection. (4) Seed trait characterization: test a subset of seeds for key traits, such as seed mass, dormancy level, and germination responses, to environmental variables like drought or temperature. (5) Long‐term conservation: place the remainder of the seed collection into long‐term storage, along with associated metadata, for future research and restoration use. (6) Regular resampling: revisit target populations at periodic intervals (depending on the species regeneration time) to collect new seeds and data, creating a dynamic temporal archive of genetic snapshots from each time point (7) spanning decades (i.e. > 50 yr).

Initiatives such as ‘The Project Baseline’ in the United States have recognized the potential of integrating temporal and spatial dimensions into seed‐collecting strategies (Etterson *et al*., 2016; Franks *et al*., 2018). ‘The Project Baseline’ has established a genetic archive intended for use over the next 50 yr (Etterson *et al*., 2016). Through this project, seeds from 100 to 200 maternal lines are being collected and stored separately from each target population across environmental gradients, creating a genetic archive intended for long‐term evolutionary studies (Etterson *et al*., 2016). Drawing inspiration from this initiative, we propose a long‐term experimental approach in which a limited number of populations of species of interest for NbS or conservation (for species whose conservation and legal protection status would allow it) are identified within the regional and taxonomic remit of a seed bank, and for which a long‐term monitoring of environmental data is coupled with seed collecting and testing, and tissue sampling for genetic comparisons, allowing for the building of historical collections of seed material with enhanced (biological and environmental) passport data, as detailed in Fig. 3. Although the number of individuals to be sampled should be identified according to population size and international guidelines for sustainable seed collecting (e.g. ENSCONET, 2009), they should not be less than eight, which is the minimum recommended for population genomics studies (Nazareno *et al*., 2017).

Data gathered from the natural populations at each time point (e.g. population genetics, plant phenology, environmental variables such as rainfall and air and soil temperature in the year of collecting) together with those on morphological and functional seed traits (e.g. seed mass, seed dormancy level, seed germination responses to temperature/drought) and seedling traits (e.g. relative growth rate and survival) will provide trends of variation over time. In addition, by targeting multiple populations per species, it will be possible to add an extra (spatial) dimension to the experimental design, enabling spatial and temporal Genotype × Environment (G × E) experiments (Anderson *et al*., 2011; Laccetti *et al*., 2024), which have been proven to be instrumental in understanding how population variation in early development phases (i.e. germination responses to temperature) can determine ecological resilience in response to environmental (and climatic) changes (Walter *et al*., 2020).

A portion of the collected seeds from each time point will also be stored long term in order to provide material to be tested in a future transgenerational common garden approach *sensu* Franks *et al*. (2018). Storing these seed lots in conservation seed banks under international standards not only will give them a unique level of ‘future‐proofing’, by making them available to future technological advancements (Stroud & Ratcliff, 2025), but also minimize any ‘invisible fraction problem’ effect (i.e. the nonrandom mortality of seeds during storage, with genes affecting seed survival also potentially affecting adult phenotypes; Grafen, 1988), guaranteeing, together with the inclusion of a refresher generation (Franks *et al*., 2018), the validity of the results of the resurrection studies (Franks *et al*., 2019).

### Recommendations for temporal repeated seed collecting programmes

The experimental approach discussed above can be applied only to a limited number of species and populations, for research purposes only, and should be complemented by wider dedicated seed collecting programmes, across multiple families and plant lineages (Carta *et al*., 2025). While sampling unbanked populations (i.e. moving along the spatial dimension; Fig. 1) remains a priority for *ex situ* conservation programmes, in some cases, this should be coupled with recollecting from previously sampled populations (i.e. moving along the temporal dimension; Fig. 1) to capture evolutionary changes and adaptation to more recent climates. This guarantees the conservation and availability of suitable genetic material for threatened species reintroduction and sustainable use of common species in NbS. In addition, as shown in the two case studies, conservation seed banks hold old collections that, even as bulked seed lots, could act as a starting point for plant adaptation and evolutionary studies.

Conservation seed banks must consider multiple factors when prioritizing collecting programmes, including current and emerging threats, areas of high biodiversity and endemism, species of high value for NbS, and often complex logistical constraints. Looking forward, seed collecting planning will increasingly need to consider areas most affected by climate change, as these regions are likely to experience the most significant shifts in species composition and ecological dynamics (Pörtner *et al*., 2021). Additionally, integrating species‐specific traits, such as drought tolerance, flowering time, and reproductive strategies, into collecting protocols can optimize the utility of these collections for both restoration and climate adaptation research. By targeting populations in climate‐vulnerable regions (Godefroid & Vanderborght, 2010) and focusing on traits that confer resilience, seed banks can play a proactive role in supporting biodiversity under rapidly changing environmental conditions. Species and populations can be prioritized for temporal recollecting to support either their conservation or their sustainable use in NbS and research, according to their level of threat and the purpose of the collections (Table 1).

**Table 1:** Priorities for repeated temporal seed collecting for conservation and use in nature‐based solutions (NbS) and research.

Category	Rationale	Target	Purpose
Populations for which only old materials (e.g. more than 20 yr) are currently available in seed banks and that still exist in nature	Old collections could be non‐ or maladapted to present and future climatic conditions (Hamilton, 1994; Schoen & Brown, 2001), making them potentially unsuitable for their reintroduction in nature (threatened species) and their use in NbS (common species)	Threatened and common species	Conservation NbS
	Availability of old material of species collected decades ago from populations still accessible for recollecting would represent a valuable baseline for carrying out resurrection ecology approach studies (e.g. Rauschkolb *et al*., 2022a,2022b)	Common species	Research
Species with short‐lived seeds	Due to the short ‘shelf life’ of these collections, repeated recollections from their natural populations should be considered an alternative to regeneration. Sampling frequency should be carefully planned, considering not only seed longevity under storage conditions and the risk of genetic erosion to those collections but also the conservation status of the natural populations	Threatened and common species	Conservation NbS
Species with a short regeneration time	Species with a short regeneration time such as annual herbs are more likely to show rapid evolution, increasing chances of maladaptation of old collections over time	Threatened species	Conservation
	Seed collections of these species make a good study system to detect and study evolution in action in common species	Common species	NbS Research
Species whose populations are distributed across variable climatic conditions (with or without population genetic data)	The availability of seed material from populations adapted to different environments provides information on the intraspecific genetic variability along the spatial axis. By adding a temporal axis, through temporal repeated collections, it would be possible to implement a bidimensional approach and provide seed materials for climate‐smart ecological restoration interventions. Availability of population genetic structure data (if available) in space and time will provide a detailed dataset towards understanding of plant adaptation and evolution to environmental changes.	Common species	NbS Research

Current collecting recommendations (Yenson *et al*., 2021), research (Etterson *et al*., 2016), and conservation (Kallow & Trivedi, 2017; Clubbe *et al*., 2020) projects already highlight the need for sampling methods that enhance the conservation of genetic diversity of targeted populations, with particular reference to sampling size and strategy, postharvest handling and storage, and, where possible, keeping maternal lines separate (van der Merwe *et al*., 2023), at least for species of conservation and ecological restoration interest. However, there are economic, logistic, and operational constraints that limit the banking of seed lots by separate maternal lines. In the case of the MSB, the additional resources required to collect and process maternal lines separately mean that this approach has mostly been applied where sample sizes are small. Where potential sample sizes are larger, difficult trade‐offs between maintaining maternal line separation and maximizing genetic diversity by sampling from a large number of individuals often arise.

Further to this forward‐looking approach, herbarium specimens or existing molecular datasets published from population‐level sampling can help reconstruct historical genetic diversity and structure patterns for comparison with present‐day target populations (Meineke *et al.*, 2018). A wide range of methods are available for sequencing historical specimens held in herbarium collections (Slimp *et al*., 2021; Rosche *et al*., 2022; Campos *et al*., 2023), moving the starting point for plant adaptation and evolutionary studies even further back along the temporal dimension. In addition, herbarium specimens – unlike seed collections – represent established genotypes which open windows into the evolutionary history of a species (Lang *et al*., 2019).

However, funding is a major constraint. While, historically, collecting priorities for seed banking have mainly been driven by species conservation status, rarity, and societal needs, here we argue that, at least for floras for which a good level of representativeness towards the spatial dimension has already been achieved in conservation seed banks, the temporal dimension should also be included among the criteria for resource prioritization. We acknowledge that the sampling and processing approaches described above are unlikely to be implemented with existing seed banks' resources, and *ad hoc* extra funds will need to be secured from research councils, local, national, and regional conservation authorities, or philanthropic foundations. Examples of success stories of *ad hoc*‐funded programmes include The Project Baseline, funded by the National Science Foundation (www.baselineseedbank.org/), and the Center for Plant Conservation for recollecting and testing seed longevity, funded by an Institute for Museum and Library Services National Leadership Grant (https://saveplants.org/seed‐longevity‐research), both in the United States.

## Conclusions

Conservation seed banks have evolved from their initial role as repositories for agricultural biodiversity to becoming essential tools for addressing the interconnected crises of biodiversity loss and climate change. Although *ex situ* conservation alone is not the answer to the Anthropocene biodiversity and climate crisis, conservation seed banks can already leverage their existing older collections for carrying out plant adaptation and evolution studies (Everingham *et al*., 2021; Rauschkolb *et al*., 2022b; Karitter *et al*., 2024, 2025). However, their potential has not been fully realized yet, and a transformative change towards a bidimensional (i.e. space and time) approach is needed. The genetic snapshots captured in *ex situ* collections offer a unique opportunity to study evolutionary processes and plant responses to environmental change. Approaches like resurrection ecology and transgenerational studies can be strongly boosted by incorporating the temporal dimension into seed banking practices. Although funding and available resources represent a major constraint, by adopting dynamic and forward‐looking strategies, these institutions can continue moving beyond preservation and provide climate‐resilient materials to actively support NbS in climate adaptation. By aligning their practices with the environmental and climatic challenges of the Anthropocene, seed banks can continue to serve as invaluable repositories of genetic diversity and resilience while playing a central role in understanding and shaping plant communities of the future.

## Competing interests

The authors declare no competing financial interests. All authors work in institutions managing conservation seed banks.

## Author contributions

EM conceived the idea and designed the research, with inputs from JV and TC. EM drafted the manuscript with SG and JV. SM collected the data, and EM and SM analysed the data. EM, SG, SM, AC, AE, TC and JV read, contributed, edited, and approved the final manuscript.

## Disclaimer

The New Phytologist Foundation remains neutral with regard to jurisdictional claims in maps and in any institutional affiliations.

## Supporting information


**Fig. S1** Map of UK populations with multiple seed collections.
**Table S1** Species with repeated seed collections from the same UK locality.
Please note: Wiley is not responsible for the content or functionality of any Supporting Information supplied by the authors. Any queries (other than missing material) should be directed to the *New Phytologist* Central Office.

## Data Availability

The data that support the findings of this study are available in the Supporting Information of this article (Table S1). Climatic data were downloaded from the chelsa website v.2.1 (https://chelsa‐climate.org; Karger *et al*., 2017; Karger *et al*., 2021).
