# Supplementary material for: Looking back to look ahead: the temporal dimension of conservation seed bank collections
Source: New Phytol. 2025 May 6;247(4):1589–98. doi: 10.1111/nph.70187 (PMC12267948; doi:10.1111/nph.70187)
Supplement: Supplementary file 1 — Fig. S1 Map of UK populations with multiple seed collections. Table S1 Species with repeated seed collections from the same UK locality. Please note: Wiley is not responsible for the content or functionality of any Supporting Information supplied by the authors. Any queries (other than missing material) should be directed to the New Phytologist Central Office. [file NPH-247-1589-s001.pdf]

New Phytologist

***New Phytologist* Supporting Information**

Article title: Looking back to look ahead: the temporal dimension of conservation seed bank collections

Authors: Efisio Mattana, Sandrine Godefroid, Stephanie Miles, Angelino Carta, Andreas Ensslin, Ted Chapman, Juan Viruel

Article acceptance date: Click here to enter a date.

The following Supporting Information is available for this article:

Fig. S1 Map of UK populations with multiple seed collections

Table S1 Species with repeated seed collections from the same UK locality**.**

Fig. S1 Map of UK populations with multiple seed collections. Distribution of the UK populations sampled more than once (within 1 km distance), with a time lag of at least 20-years and with seeds stored at the Royal Botanic Gardens, Kew Millennium Seed Bank (MSB). Yellow dot indicates the number of populations re-sampled in each area. Map created using QGIS (https://qgis.org/) and using MSB seed collections data

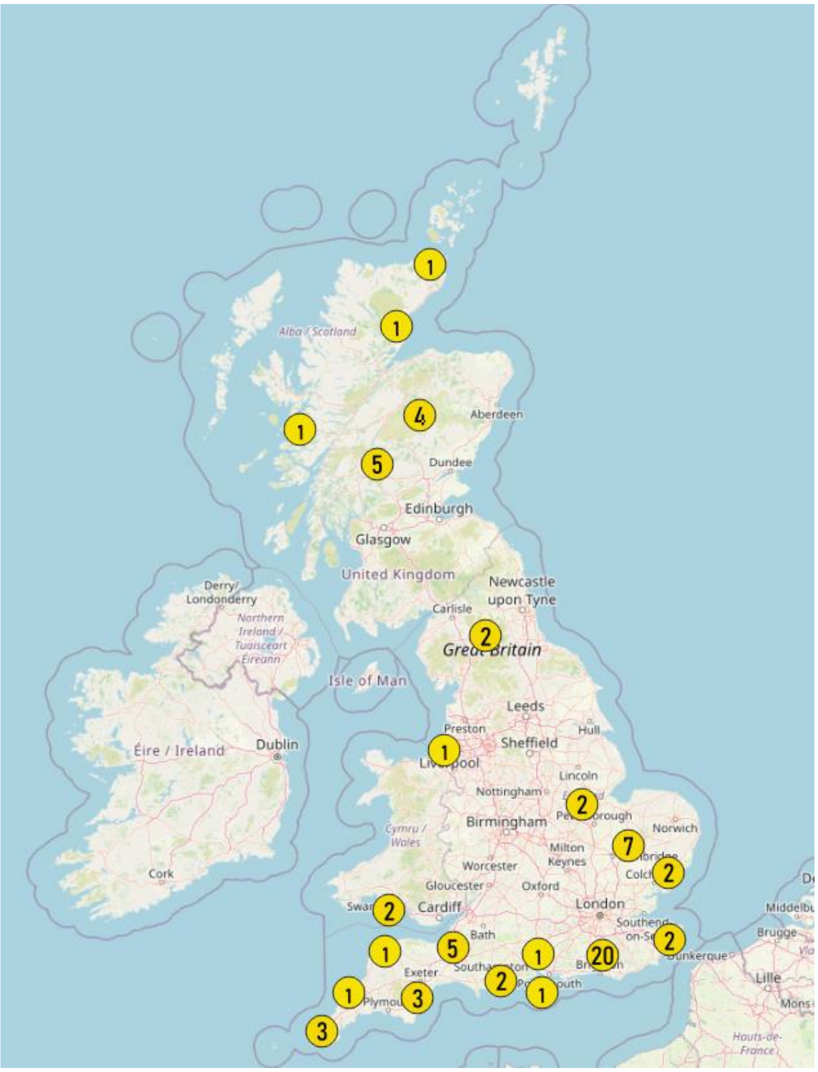

Table S1 Species with repeated collections from the same UK locality. Seed collections made with a time lag of at least 20 years (within 1 km distance), stored at the MSB.

ID	taxon	lat	long	state	region	N	from	to	range
P01	Allium schoenoprasum L.	50.01	-5.26	England	Cornwall	2	1988	2014	26
P02	Anthoxanthum odoratum L.	51.06	-0.1	England	West Sussex	3	1981	2023	42
P03	Armeria maritima subsp. elongata (Hoffm.) Bonnier	52.98	-0.54	England	Lincolnshire	4	1977	2017	40
P04	Artemisia campestris L.	52.43	0.6	England	Suffolk	3	1975	2021	46
P05	Calamagrostis scotica (Druce) Druce	58.56	-3.37	Scotland	Highlands	2	1996	2017	21
P06	Cardamine pratensis L.	51.07	-0.09	England	West Sussex	2	1999	2023	24
P07	Carex atrofusca Schkuhr	56.51	-4.58	Scotland	Tayside	2	1997	2023	26
P08	Carex buxbaumii Wahlenb.	56.89	-5.82	Scotland	Highlands	2	1996	2018	22
P09	Carex depauperata Curtis ex Woodw.	51.19	-0.63	England	Surrey	3	1992	2019	27
P10	Carex depauperata Curtis ex Woodw.	51.29	-2.8	England	Somerset	2	1991	2019	28
P11	Carex ericetorum Pollich	52.46	0.59	England	Norfolk	3	2000	2021	21
P12	Carex humilis Leyss.	51.32	-3.01	England	Somerset	2	1998	2018	20
P13	Carex lachenalii Schkuhr	57.08	-3.65	Scotland	Grampian	2	1999	2023	24
P14	Carex norvegica Retz.	56.51	-4.57	Scotland	Tayside	2	1996	2018	22
P15	Carex norvegica Retz.	56.85	-3.22	Scotland	Tayside	3	1996	2018	22
P16	Centaurea nigra L.	51.06	-0.1	England	West Sussex	2	1985	2010	25
P17	Chenopodium vulvaria L.	51.93	1.32	England	Suffolk	3	1982	2023	41
P18	Cochlearia micacea E.S.Marshall	56.54	-4.22	Scotland	Tayside	2	1994	2018	24
P19	Coincya monensis (L.) Greuter & Burdet	51.57	-4.11	Wales	West Glamorgan	2	1978	1998	20
P20	Coincya monensis (L.) Greuter & Burdet	53.42	-3.09	England	Merseyside	2	1975	2012	37
P21	Cornus suecica L.	57.13	-3.66	Scotland	Highlands	2	1997	2018	21
P22	Corrigiola litoralis L.	50.28	-3.65	England	Devon	2	1976	2017	41
P23	Damasonium alisma Mill.	51.27	-0.27	England	Surrey	3	1978	1998	20

P24	Daucus carota L.	50.88	-0.21	England	West Sussex	2	1975	2007	32
P25	Echium plantagineum L.	50.11	-5.69	England	Cornwall	2	1997	2019	22
P26	Epipactis palustris (l.) Crantz	51.56	-4.17	Wales	West Glamorgan	2	1999	2021	22
P27	Erigeron borealis Simmons	56.54	-4.23	Scotland	Tayside	2	1997	2018	21
P28	Eryngium campestre L.	50.58	-4.91	England	Cornwall	3	1993	2023	30
P29	Eryngium campestre L	51.42	0.26	England	Kent	2	1993	2015	22
P30	Euphorbia hyberna L.	50.26	-5.28	England	Cornwall	2	2002	2022	20
P31	Euphorbia platyphyllos L.	51	-2.94	England	Somerset	2	1997	2018	21
P32	Gentiana verna L.	54.7	-2.38	England	Cumbria	2	1992	2015	23
P33	Gentianella amarella (L.) Börner	50.9	-0.04	England	East Sussex	2	1981	2007	26
P34	Himantoglossum hircinum (L.) Spreng.	51.27	1.38	England	Kent	2	1998	2024	26
P35	Hyacinthoides non-scripta (L.) Chouard ex Rothm.	51.07	-0.09	England	West Sussex	2	1978	2007	29
P36	Hypericum linariifolium Vahl	50.69	-3.72	England	Devon	2	1976	1998	22
P37	Juniperus communis L.	50.98	-1.09	England	Hampshire	2	1985	2013	28
P38	Lathyrus pratensis L.	51.06	-0.1	England	West Sussex	2	1985	2010	25
P39	Linum perenne L.	52.17	0.17	England	Cambridgeshire	2	1976	1999	23
P40	Lithospermum officinale L.	50.9	-0.04	England	East Sussex	2	1981	2007	26
P41	Lobelia urens L.	50.7	-2.31	England	Dorset	3	1978	2015	37
P42	Lobelia urens L.	50.75	-1.71	England	Hampshire	3	1977	2019	42
P43	Lotus corniculatus L.	51.06	-0.1	England	West Sussex	2	1985	2010	25
P44	Luzula arcuata (Wahlenb.) Sw.	57.07	-3.67	Scotland	Central	2	1998	2018	20
P45	Malva moschata L.	51	-0.5	England	West Sussex	2	1976	2010	34
P46	Melampyrum arvense L.	50.59	-1.24	England	Isle of Wight	2	1992	2017	25
P47	Moneses uniflora (L.) A.Gray	57.95	-4.04	Scotland	Highlands	3	1990	2013	23
P48	Myosotis alpestris F.W.Schmidt	55.54	-4.23	Scotland	Tayside	3	1997	2018	21
P49	Ophrys holosericea subsp. holosericea	51.15	0.98	England	Kent	2	1999	2023	24
P50	Ophrys sphegodes Mill.	50.84	-0.05	England	East Sussex	2	1998	2022	24
P51	Orchis militaris L.	52.34	0.55	England	Suffolk	2	1998	2021	23
P52	Orobanche purpurea Jacq.	52.42	0.53	England	Suffolk	2	1991	2022	31
P53	Phyteuma orbiculare L.	50.89	-0.04	England	East Sussex	2	1981	2011	30
P54	Phyteuma spicatum L.	50.85	0.2	England	East Sussex (loc. 1)	2	1994	2014	20

P55	Phyteuma spicatum L.	50.98	0.2	England	East Sussex (loc. 2)	4	1980	2013	33
P56	Pulsatilla vulgaris Mill.	52.63	-0.41	England	Cambridgeshire	3	1999	2024	25
P57	Ranunculus acris L.	51.06	-0.09	England	West Sussex	2	1999	2023	24
P58	Ranunculus arvensis L.	52.08	1.01	England	Suffolk	2	1994	2014	20
P59	Ranunculus repens L.	51.06	-0.09	England	West Sussex	2	1975	1999	24
P60	Romulea columnae Sebast. & Mauri	50.59	-3.47	England	Devon	2	1981	2023	42
P61	Selinum carvifolia (L.) L.	52.12	0.18	England	Cambridgeshire	2	1974	1997	23
P62	Silene flos-cuculi (L.) Greuter & Burdet	51.07	-0.09	England	West Sussex	2	1969	2010	41
P63	Teucrium scordium L.	51.08	-4.21	England	Devon	3	1988	2022	34
P64	Teucrium scordium L.	52.33	0.27	England	Cambridgeshire	2	1976	2023	47
P65	Trollius europaeus L.	54.67	-2.23	England	Durham	2	1989	2013	24
P66	Valeriana rimosa (Bastard) Christenh. & Byng	50.99	-2.94	England	Somerset	3	1998	2020	22
P67	Vicia parviflora Cav.	50.99	-2.94	England	Somerset	2	1997	2018	21
